# Long-term ocular biometric variations after scleral buckling surgery in macula-on rhegmatogenous retinal detachment

**DOI:** 10.1186/s12886-021-01928-0

**Published:** 2021-04-10

**Authors:** Giuseppe Maria Albanese, Alberto Cerini, Giacomo Visioli, Marco Marenco, Magda Gharbiya

**Affiliations:** Ophthalmology Unit - Department of Sense Organs, Sapienza University of Rome – Policlinico Umberto I University Hospital, Rome, Italy

**Keywords:** Retinal detachment, Scleral buckle, Axial length, Anterior chamber depth, Biometry

## Abstract

**Background:**

Myopic shift and biometric ocular changes have been previously observed after scleral buckling (SB) surgery in rhegmatogenous retinal detachment (RRD), but long term-term outcomes had not yet been explored. The purpose of present study is to evaluate long term ocular biometric changes in patients with primary macula-on RRD treated with SB.

**Methods:**

In this retrospective, observational study, we reviewed the medical records of patients undergoing SB surgery for macula-on RRD. Ocular biometry was performed before and at the most recent visit after surgery. Axial length (AXL), anterior chamber depth (ACD), anterior corneal astigmatism and spherical equivalent in treated eyes were compared before and after surgery as well as with those of fellow eyes.

**Results:**

Thirty-four eyes of 17 patients with a mean age of 57.0 ± 8.9 years were included. The mean follow-up duration was 50.9 ± 21.9 months (median 53.0; range 12 to 82 months). A significant postoperative AXL increase of 0.83 mm and a concomitant myopic shift of 1.35 diopters was observed in the operated eyes (*p* <  0.0001). The preoperative AXL was the only predictive factor of AXL change after surgery (B = 0.152, 95% CI 0.059 to 0.245, β = 0.668, *P* = 0.003). Compared to fellow eyes, a postoperative ACD shallowing of 0.1 mm was found in operated eyes (*p* <  0.05), while there were no long-term changes of anterior corneal astigmatism.

**Conclusions:**

We show that the preoperative AXL is the only predictive factor of AXL increase after SB surgery. Scleral encircling induces a concomitant long-term shallowing of the AC, therefore fourth generation intraocular lens (IOL) power calculation formulae should be used for patients requiring cataract surgery after SB.

## Background

Primary rhegmatogenous retinal detachment (RRD) median annual incidence rate, albeit at significant differences based on ethnicity and age, is estimated at 10.5 per 100,000 people with bilateral RRD occurring in the 7.26% of cases [1]. Pars plana vitrectomy (PPV) and scleral buckling (SB) are the two principal surgical options to manage RRD and, in some selected and particularly severe cases, a combination of both procedures may be carried out. Even if several studies have tried to assess significant long-term anatomical and functional differences between the two techniques, the choice of a method at the expense of the other is not strongly supported by evidence, especially since each procedure may vary among centers and surgeons’ experience [2, 3]. Nevertheless, each surgical treatment carries potential advantages and disadvantages. Specifically, SB surgery, by compressing the eye circumferentially, is known to cause myopic shift and axial length (AXL) increase. However, most previous studies that have evaluated AXL changes following SB procedure have been limited to a maximum follow-up duration of 1 year and the long-term effects of scleral buckling on the progression of myopia are not yet clearly known [4, 5]. Indeed, circumferential compression of the eye induces structural scleral alterations as well as modifications of retinochoroidal circulation that may determine biometric changes even beyond 1 year after surgery [6]. Furthermore, ultrasound biometry was used in the majority of previous reports while only few recent studies addressed this issue by using optical biometry, which has been shown to be more accurate [7–11]. In fact, while optical biometry measures from the front of the cornea to the retinal pigment epithelium, A-scan ultrasound uses the vitreoretinal interface as posterior target for AXL measurement. This means that, by using ultrasound biometry in the setting of RRD, AXL could be underestimated by the interface of the detached retina.

In the present paper, using optical biometry, we evaluated the long-term effects of scleral encircling on the main ocular biometric parameters by comparing changes in encircled eyes with changes in the contralateral eye. To rule out any potential bias related to retinal detachment only macula-on RRD patients were included.

## Methods

We conducted an institutional cross-sectional study with fellow-eye comparison, in accordance with the tenets of the Declaration of Helsinki and approved by the ethical board of the Sapienza University of Rome. We retrospectively revised the medical charts of patients who were treated with SB for primary macula-on RRD between January 2013 and January 2018 at the Vitreoretinal Surgery Unit of the Policlinico Umberto I University Hospital of Rome.

All the selected patients were recalled and underwent ophthalmological evaluation including biometric measurements. At that time, all patients signed a written informed consent to the study.

Patients were included with the following criteria: 1) primary macula-on RRD successfully treated by SB with no further interventions, 2) a follow-up of at least 12 months after surgery, 3) age > 18 years, 4) preoperative measurements of AXL using optical biometry in both eyes, and 5) clear ocular media (opacity grade less than or equal to 2, according to the Lens Opacities Classification System III) [12].

We excluded patients with: 1) macular diseases like age-related macular degeneration, myopic choroidal neovascularization or any vitreo-macular interface alteration, e.g. macular hole or epiretinal membrane, 2) uveitis or any further disease except for non-complicated RRD, 3) history of trauma, and 4) previous ocular surgery, including refractive surgery. We also excluded patients who underwent further intraocular surgery during the follow-up period.

All SB surgeries were performed by one experienced retinal surgeon (MG). After the positioning of a 240 encircling silicone band, a silicone sleeve was used to secure its ends (Mira; Mira Inc., Uxbridge, Massachusetts, USA). All eyes underwent external SRF drainage. As part of a standardized protocol, the circumference of the 240 encircling band was shortened by 10 mm and transscleral cryotherapy was performed to the retinal break(s). A 287 circumferential silicone scleral explant was finally positioned to support the break(s) [13].

Before surgery, a complete ophthalmological evaluation including optical biometry in both eyes (IOL Master 500, Carl Zeiss Meditec, Dublin, CA) was performed and it was repeated at the last follow-up visit. The average of 5 repeated measurements per eye was used.

Preoperative and postoperative data collection included a complete medical and ophthalmic history, spherical equivalent, best-corrected visual acuity (BCVA), AXL, anterior chamber depth (ACD), anterior corneal astigmatism, lens status and intraocular pressure (IOP). Optical coherence tomography and careful binocular indirect retinal examination were assessed to evaluate the presence of macular involvement and the characteristics of the RDs.

Statistical analysis was performed using SPSS v. 25.0 (SPSS, Inc., Chicago, IL, USA). Graphs were generated using STATA, v. 14.0 (StataCorp, TX, USA). The Shapiro-Wilk test has been used to analyze normal distribution of data. Paired t –test or the Wilcoxon’s signed ranks test were used to compare longitudinal measurements in the operated eyes, as appropriate. Fisher’s exact test was used to compare categorical variables. Paired t-test or the Wilcoxon’s signed ranks test were also used to compare biometric measurements between operated and fellow eyes. Bivariate relationships were assessed by the Pearson analysis or the Spearman coefficient, as appropriate. Backward stepwise linear regression analysis was used to investigate the preoperative factors associated with AXL changes after surgery. The variables with *p* > 0.05 were removed from the model. The variables significantly associated with AXL change in the bivariate correlations as well as clinically relevant variables were included as potential prognostic factors in the regression analysis. Given the potential collinearity issue between preoperative AXL and spherical equivalent, we opted for the first to be used as an independent variable in the regression model for clinical reasons. Data were presented as mean values ± standard deviation (SD). For each value, when available, we reported estimated differences or effects, *p* values and confidence intervals (CI 95%).

## Results

According to the study inclusion criteria, we selected 51 patients of whom 34 were excluded for the following issues: 3 patients for the presence of a macular epiretinal membrane, 3 patients were affected with glaucoma, 2 patients had history of trauma, 5 patients had history of refractive surgery, 6 patients had history of phacoemulsification before RD surgery, 9 patients underwent cataract surgery during the follow-up period and 6 patients were not available to the study.

### Baseline characteristics of the cohort

We finally included a total of 34 eyes of 17 patients (9 men, 8 women) with a mean age of 57.0 ± 8.9 years (range 39 to 82). The mean follow-up duration was 50.9 ± 21.9 months (median 53.0; range 12 to 82; 95% CI 39.70 to 62.19). Patients were all phakic in both eyes. There was no difference in baseline ocular biometric measurements between RRD and fellow eyes. Before surgery, mean AXL in RRD eyes was 24.5 ± 1.0 mm (median 24.3; range 22.9 to 27.3; 95% CI 24.0 to 25.0). The preoperative clinical characteristics of RRD and fellow eyes are shown in Table 1.

**Table 1:** Baseline clinical characteristics of RRD and fellow eyes

	Operated eyes	Fellow eyes	Difference (CI 95%)	***P***-value
Duration of symptoms before RRD surgery (days), mean SD	5.12 ± 3.64			
RRD extent > 2 quadrants, n (%)	2 (12%)			
Buckle extent (clock hour), mean SD	2.5 ± 1.5			
BCVA (logMAR), mean SD	0.27 ± 0.27	0.06 ± 0.17	0.21 (0.07, 0.34)	0.006 *
AXL (mm), mean SD	24.5 ± 1.0	24.4 ± 1.0	0.08 (− 0.12, 0.28)	0.391 *
Spherical equivalent (D), mean SD	−0.96 ± 1.87	−0.79 ± 1.71	−0.16 (− 0.54, 0.21)	0.373 *
ACD (mm), mean SD	3.29 ± 0.30	3.19 ± 0.33		0.5 °
Corneal astigmatism (D), mean SD	1.29 ± 1.29	0.99 ± 0.64	0.30 (− 0.19, 0.78)	0.212 *
IOP (mmHg), mean SD	15.71 ± 1.96	15.76 ± 1.44		0.707 °

*RRD* rhegmatogenous retinal detachment, *BCVA* best corrected visual acuity, *LogMAR* logarithm of the minimum angle of resolution, *SD* standard deviation, *AXL* axial length, *ACD* anterior chamber depth, *D* diopters, *IOP* intraocular pressure, *CI* confidence intervals

* Paired t-test, ° Wilcoxon’s signed ranks test

### Biometric changes

**Fig. 1:**
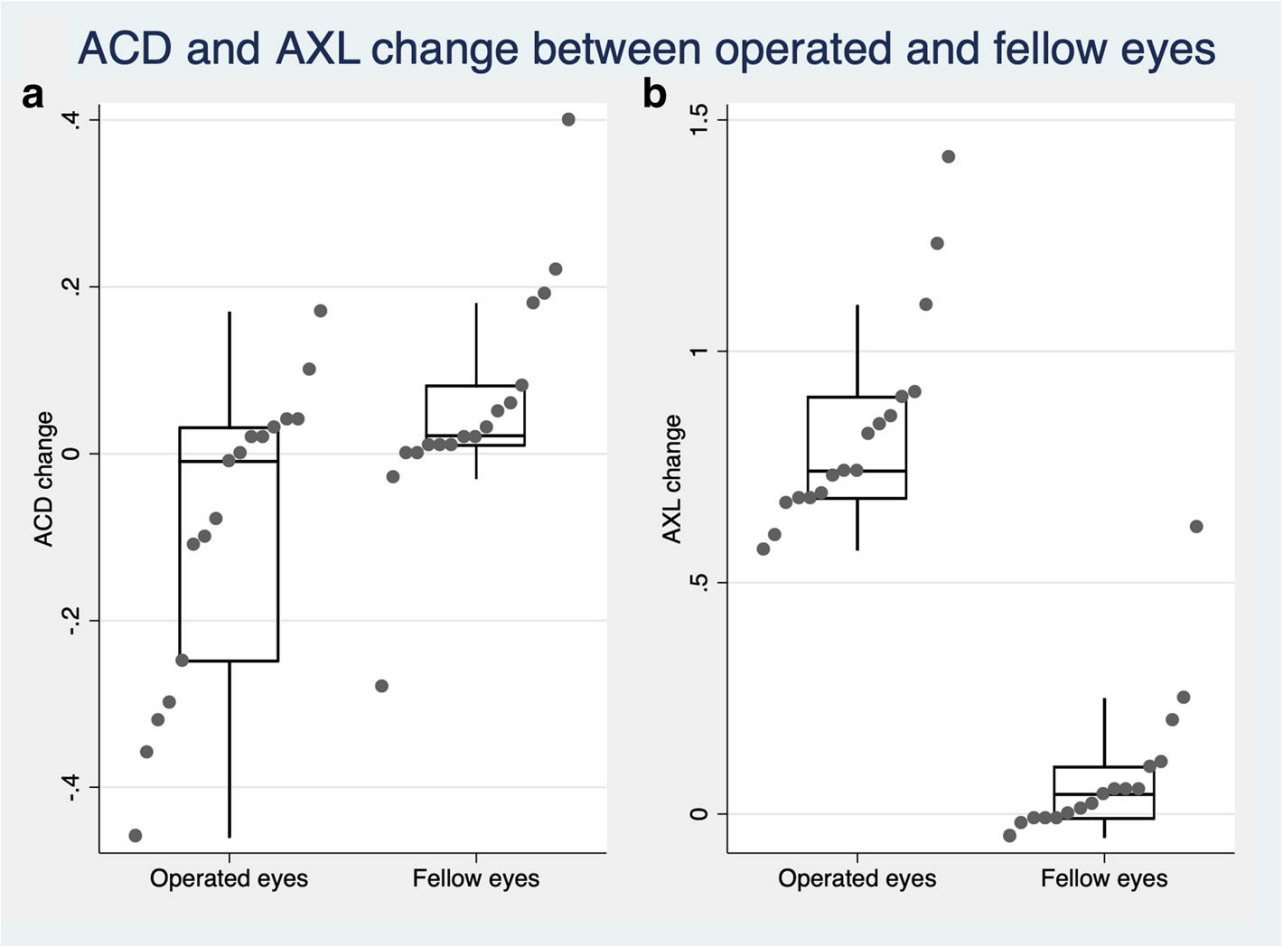
Boxplot showing **a** anterior chamber depth (ACD) and **b** axial length (AXL) change in operated and fellow eyes

Compared to baseline, at last follow-up there was a mean increase in AXL of 0.83 mm (95% CI 0.72 to 0.95, *p* <  0.0001), a mean decrease in ACD of - 0.09 (95% CI − 0.19 to 0.0007, *P* = 0.049), and a mean myopic shift of 1.35 diopters (95% CI − 1.65 to − 1.06, *p* <  0.0001). Compared to fellow eyes, AXL increased and ACD as well as spherical equivalent decreased in operated eyes (*p* <  0.05), (Fig. 1 a, b). Anterior corneal astigmatism did not differ neither from baseline nor with respect to unoperated eyes (*p* = 0.08 and *p* = 0.7, respectively). No changes in IOP were found in both operated and fellow eyes. Table 2 shows the longitudinal changes of all ocular biometric measurements in operated and fellow eyes.

**Table 2:** Postoperative results of ocular biometry of operated and fellow eyes

	Operated eye (mean ± SD)			Fellow eye (mean ± SD)			
	Baseline	Last FU	Difference (CI 95%) ***P***-value ^**a**^	Baseline	Last FU	Difference (CI 95%) ***P***-value ^**a**^	Difference (CI 95%) ***P***-value ^**b**^
AXL (mm)	24.5 ± 1.0	25.4 ± 1.2	0.83 (0.72, 0.95) < 0.0001*	24.44 ± 1.0	24.52 ± 1.2	0.08 (0.00, 0.16) 0.049*	
AXL *change*	0.83 ± 0.23			0.08 ± 0.16			0.75 (0.65, 0.85) < 0.0001*
ACD (mm)	3.29 ± 0.30	3.19 ± 0.30	−0.09 (−0.18, 0,00) 0.049*	3.19 ± 0.33	3.25 ± 0.37	0.06 (− 0.15, 0.12) 0.113*	
ACD *change*	−0.09 ± 0.18			0.06 ± 0.14			0.15 (0.06, 0.24) 0.003*
Corneal astigmatism (D)	−1.29 ± 1.3	−1.53 ± 1.2	0.08°	−1.05 ± 0.60	−1.04 ± 0.65	0.7°	
*change*	− 0.24 ± 0.43			0.02 ± 0.17			0.1°
Spherical equivalent (D)	−0.96 ± 1.87	−2.31 ± 1.92	< 0.0001°	−0.79 ± 1.71	−0.93 ± 1.70	0.3°	
*change*	− 1.35 ± 0.57			− 0.13 ± 0.45			< 0.0001°

*AXL* axial length, *ACD* anterior chamber depth, *D* diopter, *FU* follow-up, *CI* confidence interval

^a^ Comparison with baseline within the same eye

^b^ Comparison between operated and fellow eyes

* Paired t-test, ° Wilcoxon’s signed ranks test

### Other analyses

The average rate of change in AXL during the entire observation period was 0.024 ± 0.026 mm/month in the operated group and 0.002 ± 0.006 mm/month in the control group with a mean difference of 0.022 mm/month (95% CI 0.011 to 0.033, *p* <  0.0001).

**Fig. 2:**
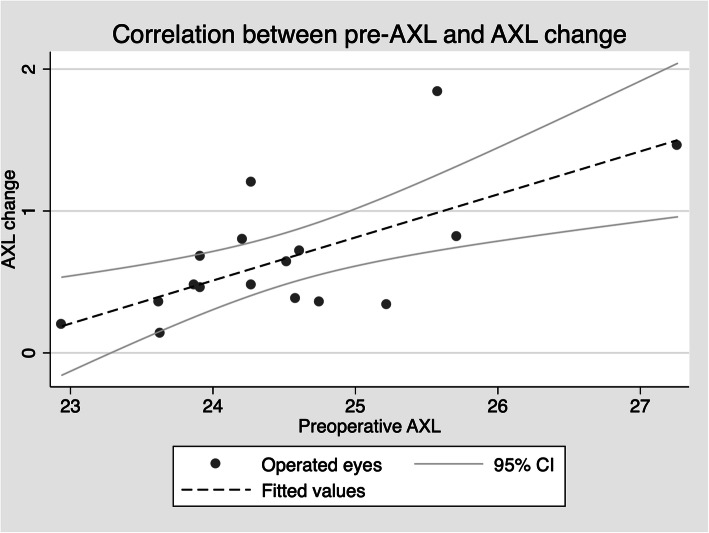
Scatterplot showing the correlation between preoperative axial length (AXL) and AXL change in mm after surgery

Taking into consideration the operated eyes, a correlation between AXL change and both the preoperative AXL (*r* = 0.668, *p* = 0.003), preoperative spherical equivalent (*r* = − 0.546, *p* = 0.02) (Fig. 2) and follow-up length (*r* = 0.521, *p* = 0.03) was found. Further, we found a weak correlation between AXL change and age (*r* = − 0.467 *p* = 0.059). AXL change was also inversely correlated to ACD change after surgery (*r* = − 0.526, *p* = 0.03). No further correlation was found.

Backward stepwise linear regression analysis showed that preoperative AXL was the only factor that independently correlated with AXL change after surgery (B = 0.152, 95% CI 0.059 to 0.245, β = 0.668, *P* = 0.003, adjusted *R*^2^ = 0.41). That is, for every 1-mm increase in baseline AXL, there was a mean AXL increase of 0.15 mm after surgery. Buckle extension (B = − 0.010, 95% CI − 0.089 to 0.070, β = − 0.062), age (B = − 0.003, 95% CI − 0.015 to 0.009, β = − 0.112) and length of follow-up (B = 0.003, 95% CI − 0.002 to 0.008, β = 0.252) were excluded during the backward elimination process.

## Discussion

The present cross-sectional study showed that, compared to unoperated fellow eyes, scleral buckling for RRD induced an AXL increase of 0.83 mm with a mean myopic shift of 1.35 diopters. We also found a concomitant decrease in ACD, while anterior corneal astigmatism was not significantly altered. Preoperative AXL was independently correlated with AXL increase after surgery. We found that for every 1-mm increase in baseline AXL, there was a mean AXL increase of 0.15 mm after surgery.

In the literature, there are only few reports that using optical biometry, addressed the issue of ocular biometric changes after scleral buckling for RRD [7–9], and there is only one study with a follow-up duration longer than 1 year [14]. Herein, we report the results of ocular biometric changes after a mean of 51 months from surgery. Our data showed that AXL changes after SB are substantially stable even in the very long term. Indeed, the reported short-term AXL increase in previous studies range from 0.58 to 1.31 mm. These differences in AXL changes among studies are probably related to the employed surgical technique or to the patients’ baseline characteristics. For instance, all previous studies included a different percentage of macula-off detachment. Although optical biometry accuracy is not theoretically affected by retinal elevation, significant underestimation of AXL measurements was however observed in RRD eyes with macular involvement [10, 14, 15]. To address this potential bias in the present study only macula-on RRD were selected.

Lee et al. in a recent retrospective investigation comparing, as in our study, AXL change between operated and fellow eyes, found an AXL increase of 1.31 mm after a mean follow-up of 26 months that did not significantly differ between myopic and highly myopic eyes [11]. On the contrary, our results showed that AXL change after surgery is strongly associated with preoperative AXL. However, in the study by Lee et al. it was not specified whether their evaluations were carried out in macula-on or macula off patients. In addition, no details were given regarding further surgical treatment performed in their cohort after RRD repair. These issues may have biased their results.

In opposition to our study, in a previous 12-month prospective study by Wong et al., a significant association between AXL increase after surgery and the extent of segmental buckling was found [9]. Of note, in their study, 9 out of 17 patients (53%) received more than 4 clock hours segmental buckling compared to 12% only (2 out of 17 patients) of our series. Therefore, this apparent discrepancy may be attributed to differences in the surgical procedure, that is the use of more extensive segmental buckling in Wong’s cohort.

Goezinne et al., in a prospective study, found transient ACD decrease after SB that returned to normal levels at 1 year after surgery [7]. In contrast, in our series we observed that, especially when comparing operated with fellow eyes, AC shallowing after SB persists in the long term - up to 51 months after surgery - suggesting that 4th generation IOL formulae that include ACD should be preferred when those patients will require cataract surgery [16]. Our results are somehow in line with two previous studies that reported significant post-SB ACD decrease up to 12 months after surgery [8, 9]. ACD decrease after scleral encircling has been attributed to the anterior rotation of ciliary body associated with surgically induced ciliary congestion and subsequent forward shift of the iris-lens diaphragm [17]. Another potential mechanism should be the low IOP prior to surgery that may induce a transient deepening of AC.

In agreement with previous reports, that described transient corneal astigmatism increase up to 3 months after SB, we did not find any clinically significant long-term postoperative change of anterior corneal astigmatism [9, 18, 19]. However, after a mean follow-up of 51 months we observed a mean change of anterior corneal astigmatism of approximately 0.25 diopters from baseline.

Among the strengths of this study we can mention the long follow-up, the fellow eye comparison as well as the choice of scrupulous inclusion and exclusion criteria. Indeed, only eyes with macula-on RRD were selected and any further surgery before and after the SB procedure either in the operated or the fellow eye was an exclusion criterion. All SB surgeries were performed by the same experienced retinal surgeon using a standardized technique that involved a fixed 10-mm shortening of the encircling band as well as the same buckle type in all cases. Among the shortcomings, it should be disclosed the small sample size and the retrospective, cross-sectional design which allowed us to compare only one time point after surgery from the enrolled cohort of patients. Long-term prospective studies could be useful to observe ocular biometry variations during time and confirm our findings.

## Conclusions

Our results show that even in the long term the principal effect of SB on ocular biometric parameters is the increase of the AXL. We found that the preoperative AXL was the only predictive factor of postoperative AXL change observed in operated eyes. Therefore, the effect of scleral encircling on refraction should be considered in combined vitrectomy and cataract procedures. Further, in patients requiring cataract surgery after scleral encircling, given the postoperative shallowing of the anterior chamber even in the long term, IOL power calculation formulae accounting for ACD should be used.

## Data Availability

The datasets generated and analyzed in the current study are not publicly available due to the institutional informed consent conditions but are available from the corresponding author on reasonable request.

## Abbreviations

SB: Scleral buckling
RRD: Rhegmatogenous retinal detachment
AXL: Axial length
ACD: Anterior chamber depth
IOL: Intraocular lens
PPV: Pars plana vitrectomy
BCVA: Best-corrected visual acuity
IOP: Intraocular pressure
